# Structural Basis and Function of the N Terminus of SARS-CoV-2 Nonstructural Protein 1

**DOI:** 10.1128/spectrum.00169-21

**Published:** 2021-06-16

**Authors:** Kaitao Zhao, Zunhui Ke, Hongbing Hu, Yahui Liu, Aixin Li, Rong Hua, Fangteng Guo, Junfeng Xiao, Yu Zhang, Ling Duan, Xin-Fu Yan, Yong-Gui Gao, Bing Liu, Yuchen Xia, Yan Li

**Affiliations:** a State Key Laboratory of Virology and Hubei Province Key Laboratory of Allergy and Immunology, Institute of Medical Virology, School of Basic Medical Sciences, Wuhan University, Wuhan, China; b Department of Blood Transfusion, Wuhan Children’s Hospital, Tongji Medical College, Huazhong University of Science & Technology, Wuhan, China; c Department of Pathogen Biology, School of Basic Medicine, Tongji Medical College, Huazhong University of Science and Technology, Wuhan, China; d Faculty of Science (Medical Science), The University of Sydney, Sydney, New South Wales, Australia; e School of Biological Sciences, Nanyang Technological University, Singapore; f BioBank, The First Affiliated Hospital of Xi’an Jiaotong University, Xi’an, China; g MRC Centre for Molecular Bacteriology and Infection, Imperial College London, United Kingdom; h Tongji-Rongcheng Center for Biomedicine, Huazhong University of Science and Technology, Wuhan, China; Fundacio irsiCaixa

**Keywords:** N terminus, Nsp1, SARS-CoV-2, crystal structure, protein translation, ribosome

## Abstract

Nonstructural protein 1 (Nsp1) of severe acute respiratory syndrome coronaviruses (SARS-CoVs) is an important pathogenic factor that inhibits host protein translation by means of its C terminus. However, its N-terminal function remains elusive. Here, we determined the crystal structure of the N terminus (amino acids [aa] 11 to 125) of SARS-CoV-2 Nsp1 at a 1.25-Å resolution. Further functional assays showed that the N terminus of SARS-CoVs Nsp1 alone loses the ability to colocalize with ribosomes and inhibit protein translation. The C terminus of Nsp1 can colocalize with ribosomes, but its protein translation inhibition ability is significantly weakened. Interestingly, fusing the C terminus of Nsp1 with enhanced green fluorescent protein (EGFP) or other proteins in place of its N terminus restored the protein translation inhibitory ability to a level equivalent to that of full-length Nsp1. Thus, our results suggest that the N terminus of Nsp1 is able to stabilize the binding of the Nsp1 C terminus to ribosomes and act as a nonspecific barrier to block the mRNA channel, thus abrogating host mRNA translation.

## INTRODUCTION

Severe acute respiratory syndrome coronavirus 2 (SARS-CoV-2), as a novel pathogen causing the coronavirus disease 2019 (COVID-19) pandemic, has been raging for more than a year and is affecting more than 200 countries and territories around the world. As of 5 February 2021, it has caused a total of 104,165,066 confirmed cases of COVID-19, including 2,265,354 deaths (https://covid19.who.int/).

Coronaviruses (CoVs) belong to the family *Coronaviridae* and subfamily *Coronavirinae* and can be classified into alphacoronavirus, betacoronavirus, deltacoronavirus, and gammacoronavirus (1). To date, seven human coronaviruses (HCoVs) have been identified, including HCoV-229E, HCoV-NL63, HCoV-OC43, SARS-CoV, HCoV-HKU1, Middle East respiratory syndrome-related coronavirus (MERS-CoV), and SARS-CoV-2 (2–9). Among them, HCoV-229E, HCoV-NL63, HCoV-OC43, and HCoV-HKU1 have relatively low virulence and mainly lead to mild respiratory disease, while SARS-CoV, MERS-CoV, and SARS-CoV-2 can cause acute respiratory distress syndrome and result in higher mortality rates.

SARS-CoV-2 belongs to the betacoronaviruses and possesses a single-stranded positive RNA genome containing 13 to 15 (12 functional) open reading frames, which are competent to encode 16 nonstructural proteins, 4 structural proteins (including spike [S], membrane [M], envelope [E], and nucleocapsid [N]), and 9 predicted accessory proteins (3a, 3b, p6, 7a, 7b, 8b, 9b, 9c, and ORF10) (10, 11).

Nonstructural protein 1 (Nsp1) of CoVs is regarded as a major virulence factor (12, 13). During virus infection, Nsp1 of MERS-CoV and SARS-CoV strongly suppresses expression of multiple host proteins by two-pronged strategies. On one hand, it binds to the host 40S ribosomal subunit and blocks protein translation. On the other hand, it induces modification of the 5′ region of capped mRNA templates and results in degradation of capped mRNA that contains the internal ribosomal entry site (IRES) (14–19). Together, both strategies ensure the abrogation of translational initiation for host proteins, including type I interferon (20). Recently, the cryo-electron microscopy (cryo-EM) structure of a complex containing the 40S ribosomal subunit and SARS-CoV-2 Nsp1 revealed that two C-terminal alpha helices, consisting of amino acid residues 154 to 160 and amino acid residues 166 to 179, bind to the mRNA entry tunnel in the ribosomal subunit (13, 21). In these two studies, the atomic structure of the Nsp1 C terminus was established with well-defined high-resolution density, while only global density of the Nsp1 N terminus was observed. Two other studies resolved the structure of the Nsp1 N terminus (the globular domain of Nsp1) at a 1.77-Å resolution (amino acids [aa] 10 to 127) (22) and at a 1.65-Å resolution (aa 13 to 127) (23). However, the biological function of the SARS-CoV-2 Nsp1 N terminus is unclear.

In this study, we determined the crystal structure of the N terminus (aa 11 to 125) of SARS-CoV-2 Nsp1 at a 1.25-Å resolution. In addition, we demonstrated that although aa 11 to 125 alone cannot directly inhibit host protein translation, this segment is necessary for Nsp1 to function. The N terminus of Nsp1 may act as a barrier to block mRNA entry of the ribosome channel or hinder the assembly of ribosomes. Our study deepens our understanding of how SARS-CoVs Nsp1 inhibits protein translation and provides a more detailed molecular basis for further targeted COVID-19 drug development.

## RESULTS

### Structure basis of the N terminus of SARS-CoV-2 Nsp1.

**FIG 1:**
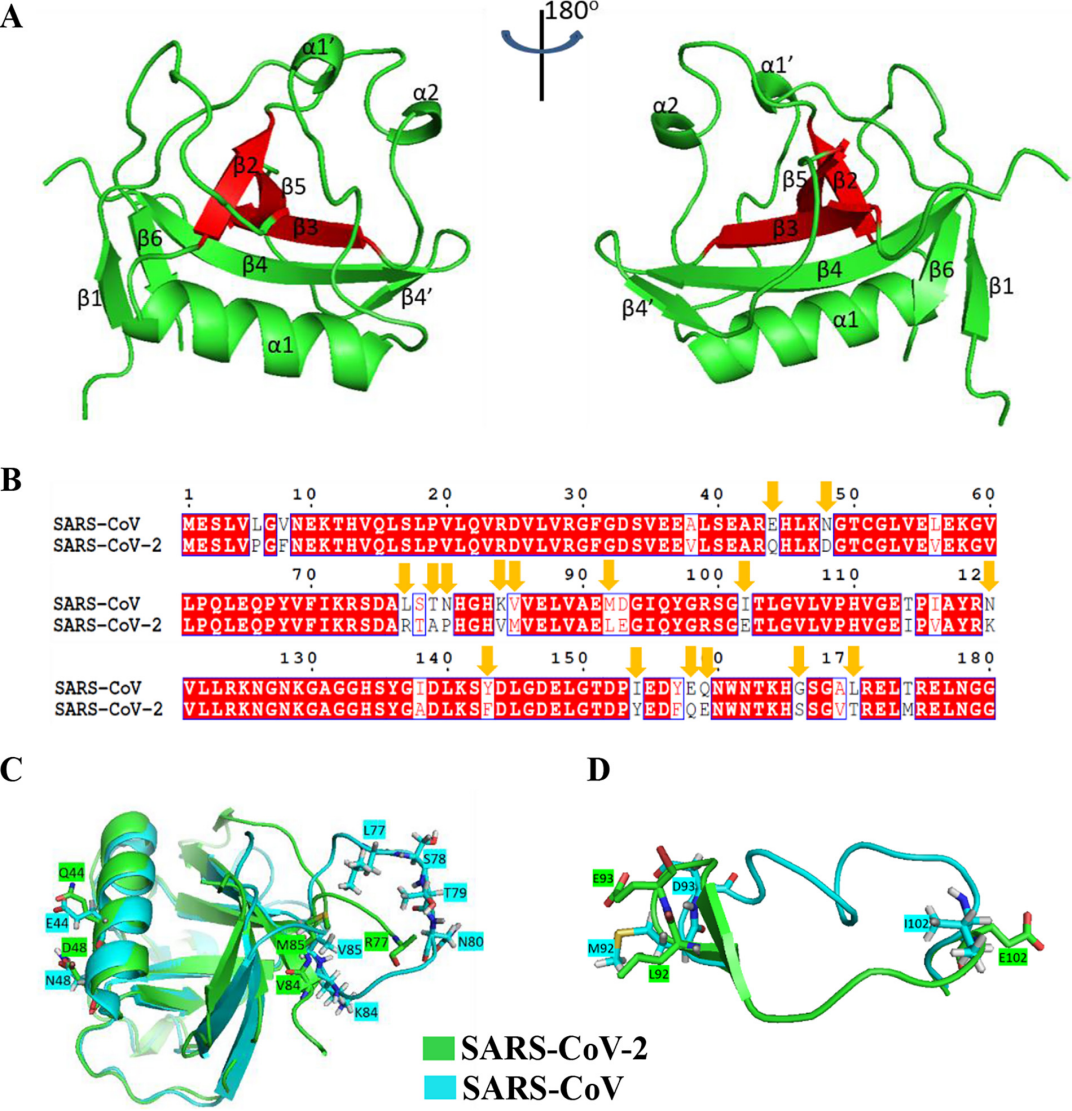
Crystal structure of SARS-CoV-2 Nsp1/11–125 and comparison with SARS-CoV Nsp1. (A) Overall crystal structure of SARS-CoV-2 Nsp1/11–125 (PDB code 7EQ4). The core of the construct (consisting of β2, β3, and β5) is in red, and the ambient barrier is in green. (B) Homology analysis between SARS-CoV-2 Nsp1 with SARS-CoV Nsp1. The residues differing in hydrophobicity or charge are highlighted with yellow arrows. (C and D) Structural alignment of SARS-CoV-2 Nsp1 with SARS-CoV Nsp1. The structural information of SARS-CoV Nsp1 was downloaded from PDB (PDB code 2HSX), and the alignment was performed using PyMOL. The different residues located at the most incompatible regions in the structure are marked and displayed as sticks.

To determine the appearance of the N terminus, we determined the crystal structure of SARS-CoV-2 Nsp1 (aa 11 to 125) at a resolution of ∼1.25 Å (PDB code 7EQ4). The overall structure consists of three α-helices and seven β-strands with the arrangement β1-α1′-α1-β2-α2-β3-β4-β4′-β5-β6 from the N terminus to the C terminus (Fig. 1A). The intersecting β2, β3, and β5 strands are arranged in a parallel or antiparallel manner, forming a core, while the β1 (residues H13 to L16), β4 (residues V84 to L92), β4′ (residues I95 to Y97), and β6 (residues V121 to R124) strands, the α1′ (residues V23 to D25), α1 (residues V35 to D48), and α2 (residues L61 to Q63) helices, and the loops (the remaining residues) surround this core, forming a closed barrel. The residues forming the core were mainly hydrophobic amino acids, among which G52, L53, and V54 lie in the β2 strand, V69, F70, and I71 form the β3 strand, and L104, G105, V106, L107, V108, and P109 form the β5 strand. Regrettably, we failed to observe the electron density of residues T78, A79, and P80 in this structure, possibly because this region is quite flexible.

As the Nsp1 of SARS-CoV and SARS-CoV-2 have similar biological functions (21), we performed structural alignment and sequence homology analysis between SARS-CoV-2 Nsp1(11–125) and SARS-CoV Nsp1(13–128) (PDB ID 2HSX). The results showed that protein sequences of Nsp1 possessed 84.44% identity between the two coronaviruses (Fig. 1B). The N-terminal structures of the two proteins were nearly the same except for two regions. The first region (residues 73 to 85) that differs between SARS-CoV and SARS-CoV-2 coincidentally contributed to the low identities in sequence (Fig. 1B and C). However, the second region (residues 93 to 102) possessed a great difference in structure with little difference in sequence identity between SARS-CoV-2 and SARS-CoV Nsp1 (Fig. 1B and D). This may in all probability be attributable to the residues at each end of the region, including L92, E93, and E102 in SARS-CoV-2, corresponding to M92, D93 and I102 in SARS-CoV.

### The N terminus is necessary for Nsp1 to inhibit protein translation.

**FIG 2:**
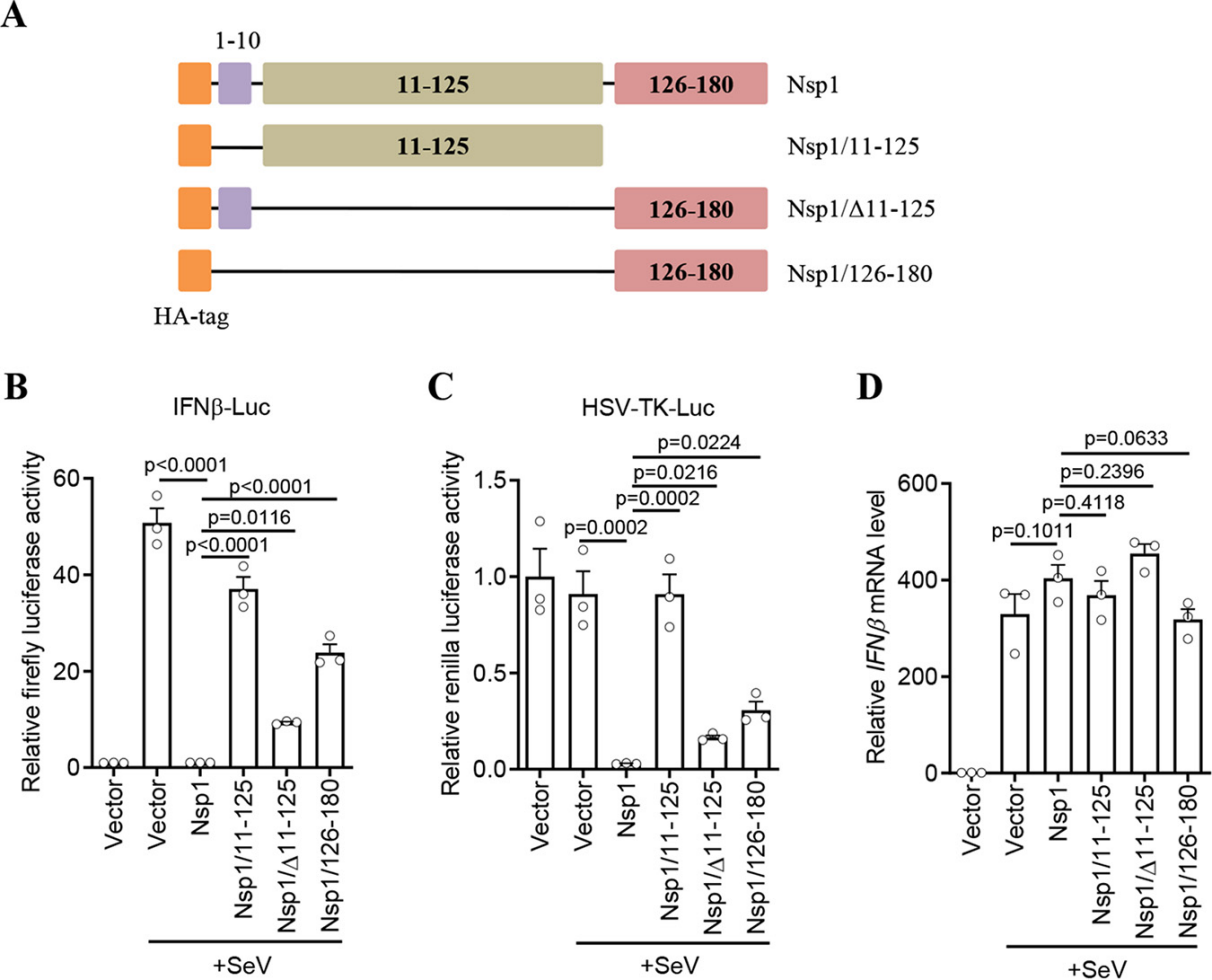
The N terminus is necessary for SARS-CoV-2 Nsp1 to inhibit protein translation. (A) Schematic diagram of SARS-CoV-2 Nsp1 and its truncated forms. (B to D) HEK293T cells (5 × 10^4^ cells/well, 48-well plates) were cotransfected with 50 ng firefly luciferase reporter plasmid for the IFN-β promoter (IFNβ-Luc), 5 ng *Renilla* luciferase reporter plasmid for herpes simplex virus thymidine kinase promoter (HSV-TK-Luc; used as an internal control), and 200 ng SARS-CoV-2 Nsp1 plasmid or its indicated truncated plasmid. SeV infection was performed at 24 h posttransfection. The reporter activity was detected with a dual-luciferase reporter system at 10 h postinfection (B and C). Total cellular RNA was extracted for mRNA analysis by real-time PCR (D). Data in panels B to D are means and standard errors of the means (SEM) from at least three independent experiments and were normalized to the control group. The *P* value was calculated in GraphPad Prism 8.2.1 using ordinary one-way analysis of variance (ANOVA) and subjected to multiple-testing correction by the false-discovery-rate (FDR) method. See also Fig. S2.

The type I interferon response has been shown to be inhibited by SARS-CoV-2 Nsp1 at the level of translation (21). To assess the role of the N terminus in the inhibition of protein translation by SARS-CoV-2 Nsp1, Nsp1/11–125 (aa 11 to 125 of Nsp1), and other Nsp1 truncated forms (Nsp1/Δ11–125 [Nsp1 missing aa 11 to 125] and Nsp1/126–180 [aa 126 to 180 of Nsp1]) were constructed (Fig. 2A). The expression and cellular localization of these proteins were detected by immunofluorescence. As shown in Fig. S1 in the supplemental material (supplemental material is found at http://gofile.me/5mkY3/HQyICSeMM) , all four proteins were successfully expressed, while Nsp1/11–125 had a higher expression level. Consistent with previous reports, the luciferase assay showed that SARS-CoV-2 Nsp1 almost completely inhibited Sendai virus (SeV)-triggered luciferase activities driven by the beta interferon (IFN-β) promoter (Fig. 2B) and herpes simplex virus thymidine kinase (HSV-TK) promoter (Fig. 2C) but had no effect on the induction of the endogenous IFN-β mRNA (Fig. 2D). These results supported the concept that SARS-CoV-2 Nsp1 specifically blocks the protein translation step (13, 21). In contrast, the Nsp1 N terminus alone (Nsp1/11–125) had almost no inhibitory effect on IFN-β promoter-mediated (Fig. 2B) or HSV-TK promoter-mediated (Fig. 2C) luciferase expression, or on IFN-β mRNA (Fig. 2D). However, when aa 11 to 125 were deleted (Nsp1/Δ11–125) or only aa 126 to 180 of Nsp1 (Nsp1/126–180) were expressed, the translational inhibition effect of Nsp1 was partially rescued but still significantly impaired compare to that of full-length Nsp1 (Fig. 2B to D). These results indicated that although aa 11 to 125 alone cannot directly inhibit protein translation, this region is necessary for Nsp1 to function. Similar results were also observed for SARS-CoV Nsp1, Nsp1/11–125, and other truncated forms (Fig. S2). Taken together, these results indicated that the N terminus of Nsp1 plays a supportive role for both SARS-CoV and SARS-CoV-2 Nsp1, and it is necessary for Nsp1 to inhibit protein translation.

### The C terminus is the key domain to ensure the colocalization between Nsp1 and ribosomes.

**FIG 3:**
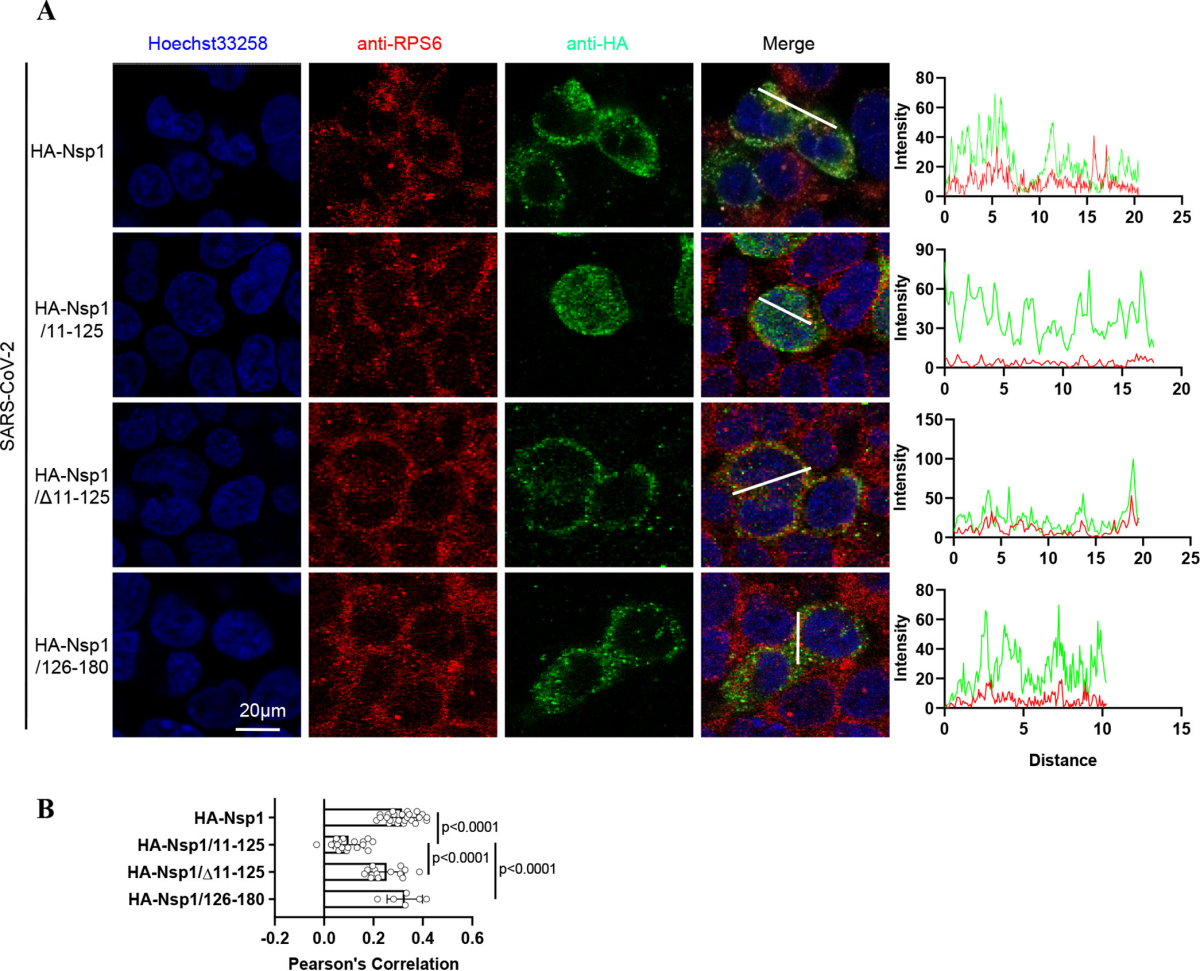
The C terminus is the key domain to ensure the colocalization between SARS-CoV-2 Nsp1 and ribosomes. (A) The colocalization of SARS-CoV-2 Nsp1 and its truncated forms (immunostained with anti-HA antibody [green]) with the ribosome (immunostained with anti-RPS6 antibody [red]) were analyzed by immunofluorescence in HEK293T cells. The intensity profile describes the distribution of Nsp1 or its truncated mutants and RPS6-specific fluorescence along the indicated line. (B) Pearson’s correlation between Nsp1 or its truncated mutants and RPS6 was analyzed and displayed. The *P* value was calculated in GraphPad Prism 8.2.1 using ordinary one-way ANOVA and subjected to multiple-testing correction by the FDR method.

As SARS-CoV-2 Nsp1 inhibits protein translation by binding to 40S ribosomal subunit (13, 21), we next investigated subcellular localization of Nsp1 and its truncated forms. As expected, Nsp1 was mainly localized in the cytoplasm and colocalized with ribosome marker ribosomal protein S6 (RPS6) (Fig. 3A and B). In contrast, Nsp1/11–125 was distributed throughout the cell and did not colocalize with ribosomes (Fig. 3A and B). Interestingly, although Nsp1/Δ11–125 and Nsp1/126–180 had less potency in translational inhibition than full-length Nsp1, they mainly localized in the cytoplasm and colocalized with ribosomes (Fig. 3A and B). Similar results were observed with SARS-CoV Nsp1 and its truncated forms (Fig. S3A and B). Overall, these results suggested that the C terminus of Nsp1 plays a vital role in ensuring the colocalization between Nsp1 and ribosomes.

### The N-terminal sequence of Nsp1 is not specific for translation inhibition.

**FIG 4:**
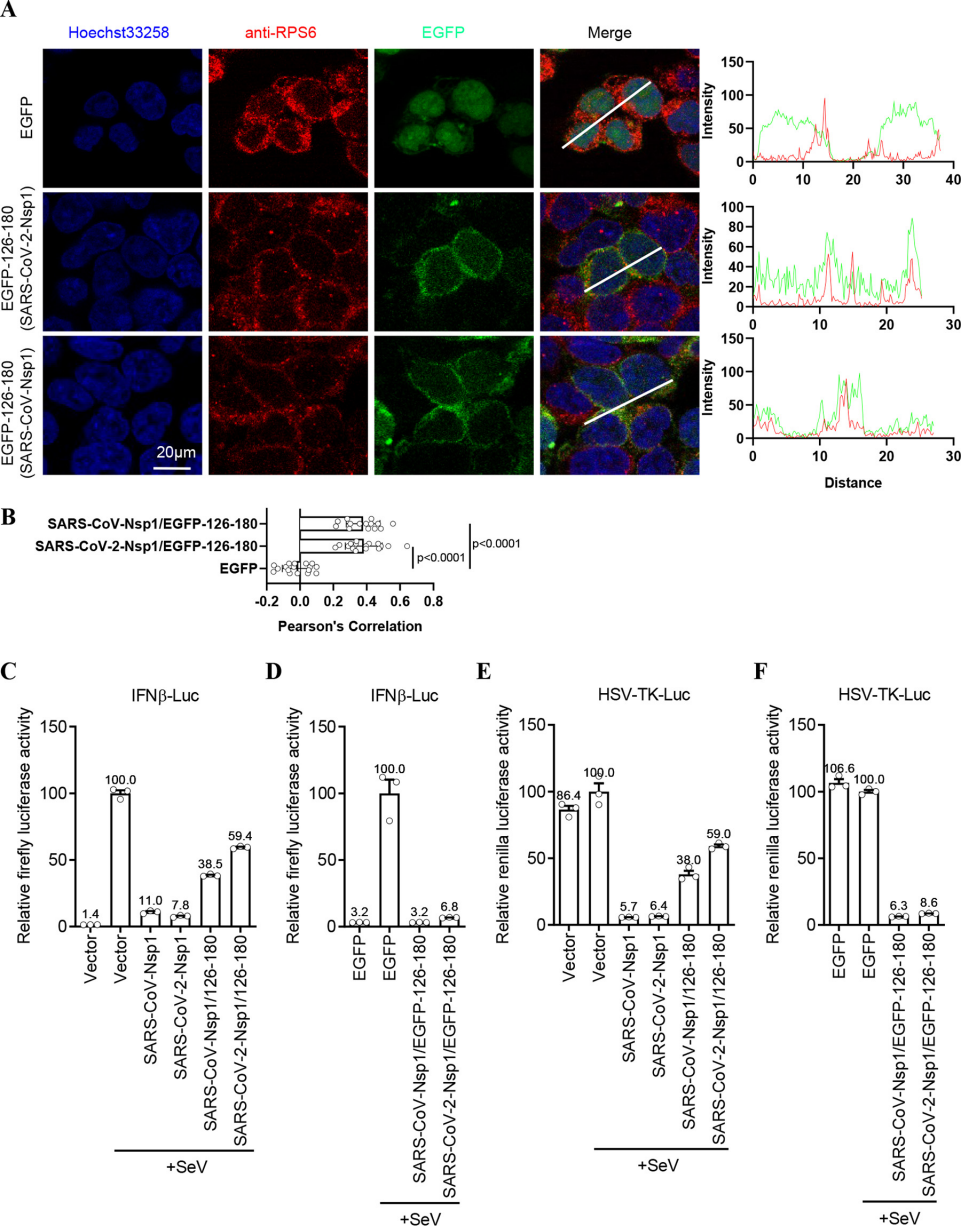
The N-terminal sequence of Nsp1 is not specific for translation inhibition. (A) The colocalization of EGFP and EGFP-fused Nsp1/126–180 (green) with ribosome (immunostained with anti-RPS6 antibody [red]) was analyzed by immunofluorescence in HEK293T cells. The intensity profile describes the distribution of EGFP or EGFP-fused Nsp1/126–180 and RPS6-specific fluorescence along the indicated line. (B) Pearson’s correlation between Nsp1 or its truncated mutants and RPS6 was analyzed and displayed. The *P* value was calculated in GraphPad Prism 8.2.1 using ordinary one-way ANOVA and subjected to multiple-testing correction by FDR method. (C to F) HEK293T cells (5 × 10^4^ cells/well, 48-well plates) were cotransfected with 50 ng firefly luciferase reporter plasmid for IFN-β promoter (IFNβ-Luc), 5 ng *Renilla* luciferase reporter plasmid for the herpes simplex virus thymidine kinase promoter (HSV-TK-Luc; used as an internal control), and 200 ng of the indicated truncated plasmid or EGFP fusion-expressing plasmid. SeV infection was performed at 24 h posttransfection. The reporter activity was detected by dual-luciferase reporter system at 10 h postinfection. Data in panels C to F are means and SEM from at least three independent experiments and were normalized to the control group.

As demonstrated above, although the C terminus was the determinant, the N terminus of Nsp1 is still necessary for the translation inhibition effect of Nsp1 (Fig. 2 and Fig. S2). However, the results also indicated that the N terminus of Nsp1 does not play a role in the colocalization between Nsp1 and ribosomes (Fig. 3 and Fig. S3). Thus, we speculated that the N terminus of Nsp1 may act as a barrier to block mRNA entry into the ribosome channel. To further verify this hypothesis, we replaced the N terminus of Nsp1 with enhanced green fluorescent protein (EGFP), a widely used fusion tag for analysis of protein dynamics and localization (24), forming the EGFP-C terminus (aa 126 to 180) fusion protein (named Nsp1/EGFP-126–180). As the results show, EGFP alone was distributed in whole cells, but the C terminus of SARS-CoV or SARS-CoV-2 Nsp1 caused the EGFP fusion protein to shift to the cytoplasm and colocalize with ribosomes (Fig. 4A and B). These results further indicated that the C terminus of Nsp1 is the key domain ensuring its binding with ribosomes. Furthermore, the luciferase reporter assay showed that while full-length Nsp1 of SARS-CoV or SARS-CoV-2 could block about 90% of IFN-β promoter-mediated luciferase activity, the C terminus of Nsp1 could suppress only 40 to 60% of the signal (Fig. 4C). Interestingly, the C terminus of Nsp1 fused with EGFP restored the ability to inhibit protein translation, which was almost equal to that of full-length Nsp1 (Fig. 4D). Similar results were also observed with the HSV-TK-mediated luciferase assays (Fig. 4E and F). To further verify this phenomenon, we also replaced the N terminus of Nsp1 with other proteins, including mCherry, large NanoBiT subunit (LgBiT), and puromycin *N*-acetyltransferase (PuroR) (Fig. S4). Interestingly, all these fusion proteins showed inhibitory effects on translation similar to that of full-length Nsp1 (Fig. S4). Unexpectedly, the fusion of tdTomato, which is larger than other fusion proteins, with the Nsp1 C terminus resulted in the ability to colocalize with ribosomes but did not restore the protein’s inhibition ability (Fig. S5). These results suggested that proper size of the Nsp1 N terminus may be important for inhibition of protein translation. In addition, these proteins have no homology or similarity with the N terminus of Nsp1 (Fig. S6). Thus, these results demonstrated that although the N-terminal sequence of Nsp1 is not specific for translation inhibition, it plays the role of a barrier outside the entrance for mRNA on the ribosome during protein translation.

**FIG 5:**
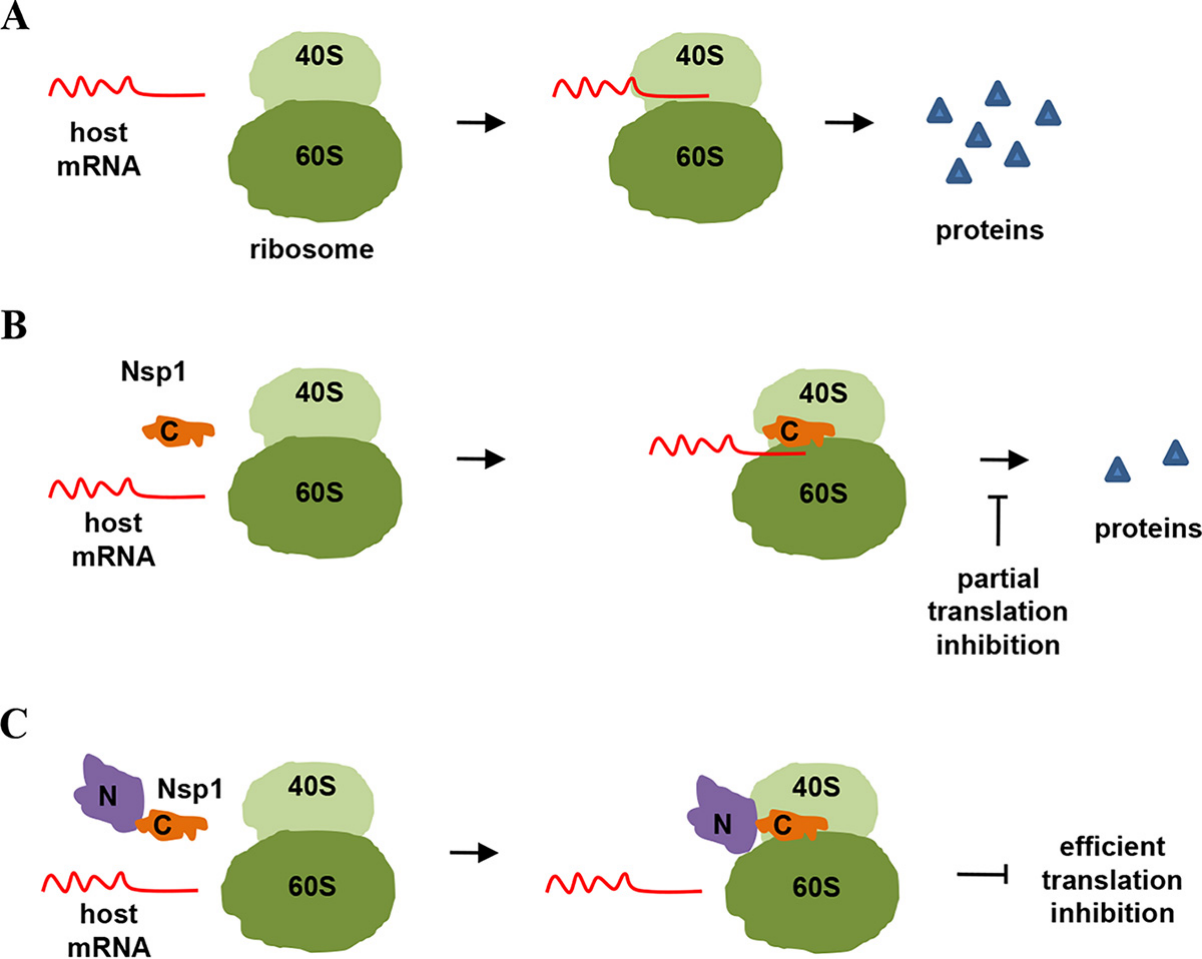
Schematic diagram of Nsp1 inhibiting protein translation. (A) In the absence of Nsp1, host mRNAs can bind to ribosomes and perform protein translation. (B) The C terminus of Nsp1 can binds to 40S ribosome and enters the mRNA entry tunnel, thus interfering with host mRNA translation to a certain extent. (C) In the presence of full-length Nsp1, the C terminus of Nsp1 first binds to 40S ribosome and enters the mRNA entry tunnel; the N terminus of Nsp1 is then pulled to the vicinity of the ribosomes and acts as an obstacle to block the entrance of the mRNA entry channel.

In summary, as presented in Fig. 5, our results showed that the C terminus of SARS-CoVs Nsp1 is the key domain to ensure the localization and function of the Nsp1, while the N terminus can act as a barrier to block the mRNA channel and thus efficiently abrogate host mRNA translation. Our study deepens the understanding of how SARS-CoVs Nsp1 inhibiting host protein translation and provides a more detailed molecular basis for further targeted COVID-19 drug development.

## DISCUSSION

Nsp1 is regarded as a major virulence factor of SARS-CoVs (12, 13). Previous cryo-EM mapping revealed that Nsp1 can inhibit protein translation by binding to the 40S ribosomal subunit though its C-terminal domain. Since the N terminus of Nsp1 had a low resolution, its functions were not well described (21). In this study, we determined the crystal structure of the N terminus (aa 11 to 125) of SARS-CoV-2 Nsp1 at a resolution of ∼1.3 Å, which has a structural basis similar to that of SARS-CoV Nsp1 (Fig. 1). To uncover the N-terminal functions, a series of truncated forms of Nsp1 were constructed. The results clearly showed that although the N terminus alone cannot directly inhibit protein translation, it is necessary for Nsp1 to inhibit protein translation (Fig. 2 and Fig. S2). Confocal analysis found that the N terminus alone lost the ability to colocalize with ribosomes, while the C terminus alone can colocalize with ribosomes (Fig. 3 and Fig. S3). In view of the fact that the C terminus alone had a significantly weaker ability to inhibit protein translation than full-length Nsp1 (Fig. 2 and Fig. S2), the idea of fusing other proteins at the C terminus to replace the N terminus was raised. Interestingly, the C terminus of Nsp1 fused with other proteins or peptides (EGFP, mCherry, LgBiT, and PuroR) exhibits almost completely restored ability to inhibit protein translation (Fig. 4 and Fig. S4), indicating that the N terminus was necessary but not specific for translation inhibition mediated by Nsp1. Thus, a model of Nsp1 inhibition of protein translation is proposed as below: First, the C terminus of Nsp1 binds to the 40S ribosome and enters the mRNA entry tunnel; second, the N terminus of Nsp1 is pulled into the vicinity of the ribosomes, acting as an obstacle to block the entrance of the mRNA entry channel.

A previous study showed that Nsp1 disrupted the binding of eukaryotic initiation factor 3 j subunit (eIF3j) to the 40S ribosomal subunit, probably due to the potential interaction between the N terminus of Nsp1 and ribosomal protein S3 (uS3), which potentially shielded access to uS3 and the mRNA-binding channel and/or made the conformation of the 40S subunit unfavorable for eIF3j interaction (13). Consistent with our conclusion that the C terminus was the key domain to ensure the colocalization between Nsp1 and ribosomes (Fig. 3 and Fig. S3), Yuan et al. showed that the binding of Nsp1 to the 40S subunit was not affected even when eIF3j was present in excess (13). These data suggested that the N terminus of Nsp1 was not essential for Nsp1 to binding to the 40S subunit. However, it may hinder further assembly of ribosomes, like the eIF3j interaction.

In a recent study, which did not include expressing the C terminus alone, Schubert et al. concluded that the N terminus did not play an important role in the inhibition of translation (25). However, our results proved that the sequence of N terminus is not specific but is necessary for Nsp1 to inhibit protein translation, based on the following observations: (i) the N terminus alone does not have the ability to inhibit protein translation (Fig. 2 and Fig. S2); (ii) although the C terminus alone can colocalize with ribosomes (Fig. 3 and Fig. S3), the ability to inhibit protein translation was significantly weaker than that of full-length Nsp1 (Fig. 2 and Fig. S2); (iii) fusing other proteins at the C terminus to replace the N terminus can restore the ability to inhibit protein translation (Fig. 4 and Fig. S4).

SARS-CoV Nsp1 effectively suppresses the translation of host mRNAs, but the SARS-CoVs themselves can overcome the Nsp1-mediated translation suppression, which was thought to be due to the 5′ untranslated region (5′ UTR) of coronavirus mRNA (14, 15). A recent study reported that the N terminus of Nsp1 directly interacted with the 5′ UTR of SARS-CoV-2 mRNA to evade translation inhibition (26). It is worth noting that a histidine–maltose-binding protein (His-MBP) tag (total molecular weight of about 70 kDa according to SDS-PAGE) was fused at the N terminus of Nsp1 and its truncated forms in that study (26). Our results suggest that only an N terminus with the proper size could be conducive to Nsp1 functioning (Fig. S5). Hence, His-MBP may be too large to function. In addition, another study reported that Nsp1 can equally inhibit the translation of reporter mRNA containing a native 5′ UTR or a SARS-CoV-2 5′ UTR (25). The highly efficient translation of SARS-CoV-2 transcripts mediated by its 5′ UTR may partly explain how the viral mRNA translation avoids being inhibited (25). How Nsp1 chooses between host mRNA and viral RNA for the inhibition of protein translation is indeed an interesting question. The N terminus of Nsp1 may play a role in this process, although controversy on this point exists (25, 26).

The COVID-19 pandemic has lasted for more than a year, and the genome mutations of SARS-CoV-2 are creating more challenges to combating the pandemic (27, 28). Recently, a series of Nsp1 mutations, especially deletion mutations, have been reported to affect the function of Nsp1 and the clinical phenotype of COVID-19 (29, 30). The deletion of 9 nucleotides in position 686 to 694, corresponding to amino acid positions 141 to 143 (KSF) at the C terminus of Nsp1, may affect the function of Nsp1 (29). In addition, a deletion in the Nsp1-coding region (Δ500–532) resulting in an 11-amino-acid deletion (aa 79 to 89) in the N terminus was found in more than 20% of sequenced samples and in 37 countries worldwide, which is associated with higher reverse transcription-PCR cycle thresholds in SARS-CoV-2 tests and lower serum IFN-β levels in infected patients (30), indicating that the N terminus of Nsp1 not only is essential for the function of Nsp1 but may also play an important role in the viral replication and disease outcome of COVID-19.

## MATERIALS AND METHODS

### Cell culture and plasmid transfection.

HEK293T cells (ATCC; CRL-3216) were obtained from the American Type Culture Collection and cultured in Dulbecco’s modified Eagle’s medium (DMEM; Gibco) supplemented with 10% fetal bovine serum (FBS; Lonza) and 100 U/ml penicillin/streptomycin (Gibco) at 37°C in a 5% CO_2_ incubator. Plasmids were transfected into cells using PEI MAX 40K (Polysciences) following the manufacturer’s instructions.

### Plasmid construction.

The sequences of SARS-CoV-2 Nsp1 and SARS-CoV Nsp1 were amplified from pcDNA6B-nCoV-Nsp1-FLAG and pcDNA6B-SARS-Nsp1-FLAG (kindly provided by Peihui Wang), respectively, and subsequently cloned into the mammalian expression vector pXJ40-HA. The deletion-containing and truncated forms of Nsp1 were constructed by a PCR-based approach. The pEGFP-C1 was purchased from Clone Tech (Somis, CA). The sequences of mCherry (from pmCherry-N1) (Clontech; catalog no. 632523), LgBiT (from pBiT1.1-N TK/LgBiT vector) (Promega; catalog no. N198A), PuroR (from pLKO.1) (Addgene; catalog no. 10878), and tdTomato (from pX330-tdTomato) were cloned and ligated to pEGFP-C1 to replace EGFP, respectively forming pmCherry-C1, pHA-LgBiT-C1, pHA-PuroR-C1, and ptdTomato-C1. The 126–180 sequence of SARS-CoV Nsp1 and SARS-CoV-2 Nsp1 was then cloned into pEGFP-C1, pmCherry-C1, pHA-LgBiT-C1, pHA-PuroR-C1, and ptdTomato-C1 to form a series of plasmids expressing the indicated fusion proteins. The IFN-β promoter–firefly luciferase reporter plasmid was purchased from Clone Tech (Somis, CA), and the herpes simplex virus thymidine kinase promoter–*Renilla* luciferase reporter plasmid (pRL-TK) was purchased from Promega (San Luis Obispo, CA). To determine the structure, the gene sequence coding SARS-CoV-2 Nsp1(11–125) was amplified and subcloned into the pET30a(+) vector with an N-terminal His_6_ tag.

### Protein expression and purification.

The recombinational plasmid PET30a(+)-Nsp1(11–125) of SARS-CoV-2 was transferred into Escherichia coli BL21(DE3) cells. A single colony was inoculated into lysogeny broth (LB) medium containing 50 μg/ml kanamycin and cultured at 37°C until the optical density at 600 nm (OD_600_) reached 0.8. IPTG (isopropyl-β-d-thiogalactopyranoside; 0.5 mM) was added to the medium, and protein expression was induced at 25°C for 16 h. Cells were harvested by centrifugation and suspended in lysis buffer containing 30 mM Tris-HCl (pH 8.0), 5% glycerol, 150 mM NaCl, 10 mM imidazole and homogenized with a high-pressure cell-crushing apparatus. The supernatant was separated by centrifugation (25,000 × *g*, 30 min) and loaded onto a 3-ml nickel-nitrilotriacetic acid (Ni-NTA) column with a flow rate of 1 ml/min. After washing with washing buffer (30 mM Tris-HCl [pH 8.0], 5% glycerol, 150 mM NaCl, 30 mM imidazole), the column was eluted with elution buffer (30 mM Tris-HCl [pH 8.0], 5% glycerol, 150 mM NaCl, 300 mM imidazole). Protein was further purified using gel filtration chromatography (Superdex 75 10/300 GL). The protein was stored in buffer A containing 30 mM Tris-HCl (pH 7.5), 150 mM NaCl, 2 mM TCEP [Tris(2-carboxyethyl)phosphine hydrochloride], 5% glycerol.

### Crystallization, data collection, and structure determination.

SARS-CoV-2 Nsp1(11–125) was crystallized via the sitting-drop vapor diffusion method at 16°C. Optimal crystals were obtained in the optimal solution containing 0.2 M lithium sulfate monohydrate, 0.1 M glycine (pH 9.5), and 30% (wt/vol) polyethylene glycol 4000. A single crystal was soaked in protectant containing 0.2 M lithium sulfate monohydrate, 0.1 M glycine (pH 9.5), 30% (wt/vol) polyethylene glycol 4000, and 20% glycerol for 1 min and rapidly cryopreserved in liquid nitrogen. Diffraction data collection was performed at beamline 17U1 of the Shanghai Synchrotron Radiation Facility (SSRF). iMosflm was used to process the diffraction data (31). Molecular replacement by Phaser was processed for the primary phase using the model of SARS-CoV Nsp1 (PDB code 2HSX) (32). Refmac and Phenix.refine were utilized for the refinement (33, 34). The final model was generated by manual model building with Coot (35). Data collection and structure refinement statistics are summarized in Table S1.

### Homologous modeling, sequence, and structure alignment.

All the sequences of coronaviruses were downloaded from GenBank. The sequence alignment was performed using the online tools ClustalW from the GenomeNet website (https://www.genome.jp/tools-bin/clustalw), and the results were exported with the online tool ESPript 3.0 (http://espript.ibcp.fr/ESPript/cgi-bin/ESPript.cgi). The released structures of Nsp1 from different coronaviruses were downloaded from Protein Data Bank (PDB), and the views were exported by PyMOL.

### Luciferase assay.

The dual-luciferase reporter assay system (E1960; Promega, San Luis Obispo, CA) was used for luciferase assays as described previously (36, 37). Briefly, cells were seeded in 48-well plates (5 × 10^4^ cells per well) and transfected with luciferase reporter (50 ng/well) and pRL-TK (5 ng/well) plasmids combined with target plasmid or empty control plasmid (200 ng/well) for 24 h. Subsequently, cells were infected with Sendai virus (SeV) for 10 h, and the luciferase activity was then measured.

### Reverse transcription–real-time PCR.

Total cellular RNA was extracted with an Ultrapure RNA kit (CW0581M; CoWin Biosciences, China) according to the manufacturer’s instructions. Total RNA (500 ng) was reverse transcribed into cDNA (Toyobo) and then analyzed by real-time PCR with FastStart Essential DNA Green Master (Roche). The following primers were used: for human IFN-β, the sense primer was 5′-AGGACAGGATGAACTTTGAC-3′ and the antisense primer was 5′-TGATAGACATTAGCCAGGAG-3′; for human β-actin, the sense primer was 5′-ATCGTGCGTGACATTAAGGAG-3′ and the antisense primer was 5′-GGAAGGAAGGCTGGAAGAGT-3′. The data were normalized to the level of β-actin mRNA in each individual sample. The 2^−ΔΔ^*^CT^* method was used to calculate relative expression changes.

### Immunofluorescence assay.

HEK293T cells were seeded at approximately 30% confluence in a confocal dish and transfected with the indicated plasmids. Indirect immunofluorescence staining of transfected cells was performed at 48 h after transfection as described previously (38). Antihemagglutinin (anti-HA) (Sigma; h6908) and anti-RPS6 (Proteintech; 66886-1-1g) were used as the primary antibodies; Alexa Fluor 488-conjugated antibody and Alexa Fluor 568-conjugated antibodies (Life Technologies) were used as the secondary antibodies. The nuclei were stained with Hoechst 33258 (Life Technologies). The cells were then analyzed using a confocal laser scanning microscope (TCS SP8 STED; Leica, Germany).

### Data availability.

All data generated or analyzed during this study are included in this article and the supplemental material. Constructs are available either through a public repository or via requests to the corresponding authors.
