# Erratum for Zhao et al., “Structural Basis and Function of the N Terminus of SARS-CoV-2 Nonstructural Protein 1”

**DOI:** 10.1128/spectrum.01002-21

**Published:** 2021-08-18

**Authors:** Kaitao Zhao, Zunhui Ke, Hongbing Hu, Yahui Liu, Aixin Li, Rong Hua, Fangteng Guo, Junfeng Xiao, Yu Zhang, Ling Duan, Xin-Fu Yan, Yong-Gui Gao, Bing Liu, Yuchen Xia, Yan Li

**Affiliations:** a State Key Laboratory of Virology and Hubei Province Key Laboratory of Allergy and Immunology, Institute of Medical Virology, School of Basic Medical Sciences, Wuhan University, Wuhan, China; b Department of Blood Transfusion, Wuhan Children’s Hospital, Tongji Medical College, Huazhong University of Science & Technology, Wuhan, China; c Department of Pathogen Biology, School of Basic Medicine, Tongji Medical College, Huazhong University of Science and Technology, Wuhan, China; d Faculty of Science (Medical Science), The University of Sydney, Sydney, New South Wales, Australia; e School of Biological Sciences, Nanyang Technological University, Singapore; f BioBank, The First Affiliated Hospital of Xi’an Jiaotong University, Xi’an, China; g MRC Centre for Molecular Bacteriology and Infection, Imperial College London, United Kingdom; h Tongji-Rongcheng Center for Biomedicine, Huazhong University of Science and Technology, Wuhan, China

## ERRATUM

Volume 9, issue 1, e00169-21, https://doi.org/10.1128/Spectrum.00169-21, 2021. In the originally published version, a supplemental material file was posted and a list of supplemental material appeared on p. 10. On 14 July 2021, the article was reposted with the following changes: the supplemental material file was removed, and a URL for supplemental data (http://gofile.me/5mkY3/HQyICSeMM) was provided on p. 3, line 6.

